# Industrial Transfer Learning for Multivariate Time Series Segmentation: A Case Study on Hydraulic Pump Testing Cycles

**DOI:** 10.3390/s23073636

**Published:** 2023-03-31

**Authors:** Stefan Gaugel, Manfred Reichert

**Affiliations:** 1 Bosch Rexroth AG, 89081 Ulm, Germany; 2 Institute of Databases and Information Systems, Ulm University, 89081 Ulm, Germany

**Keywords:** time series segmentation, deep learning, multivariate time series, transfer learning, end-of-line testing

## Abstract

Industrial data scarcity is one of the largest factors holding back the widespread use of machine learning in manufacturing. To overcome this problem, the concept of transfer learning was developed and has received much attention in recent industrial research. This paper focuses on the problem of time series segmentation and presents the first in-depth research on transfer learning for deep learning-based time series segmentation on the industrial use case of end-of-line pump testing. In particular, we investigate whether the performance of deep learning models can be increased by pretraining the network with data from other domains. Three different scenarios are analyzed: source and target data being closely related, source and target data being distantly related, and source and target data being non-related. The results demonstrate that transfer learning can enhance the performance of time series segmentation models with respect to accuracy and training speed. The benefit can be most clearly seen in scenarios where source and training data are closely related and the number of target training data samples is lowest. However, in the scenario of non-related datasets, cases of negative transfer learning were observed as well. Thus, the research emphasizes the potential, but also the challenges, of industrial transfer learning.

## 1. Introduction

### 1.1. Problem Statement

The ubiquity of sensor data in modern manufacturing is creating opportunities as well as challenges for manufacturers. Since both computer-aided engineering [1] and time series-based machine learning (ML) have shown significant advancements in recent years, an increasing number of manufacturers rely on ML-based solutions to create value out of sensor data. In particular, fields like predictive maintenance [2], condition monitoring [3], and anomaly detection [4] have achieved immense progress and have become well-known terms in the academic and industrial world.

A lesser-known research area is time series segmentation (TSS), which deals with splitting up a time series into distinct non-overlapping segments. In a manufacturing setting, TSS is usually based on multivariate time series data. TSS can be utilized to detect different operational states of production machines or to recognize human actions via tracking sensors. Multivariate time series data pose special challenges for algorithms. The most successful multivariate TSS approaches proposed in recent years are based on deep learning and use a supervised learning concept (e.g., [5]). In recent work, we analyzed a TSS use case in manufacturing and proposed a novel deep learning architecture [6]. The presented network was successful in splitting up the end-of-line testing cycle data of hydraulic pumps into different testing phases based on nine sensor channels. However, results were based on a sufficiently large training dataset that was available for each pump variant. In this paper, we build on this previous work and revisit the use case with the difference that we face training data scarcity.

**Figure 1:**
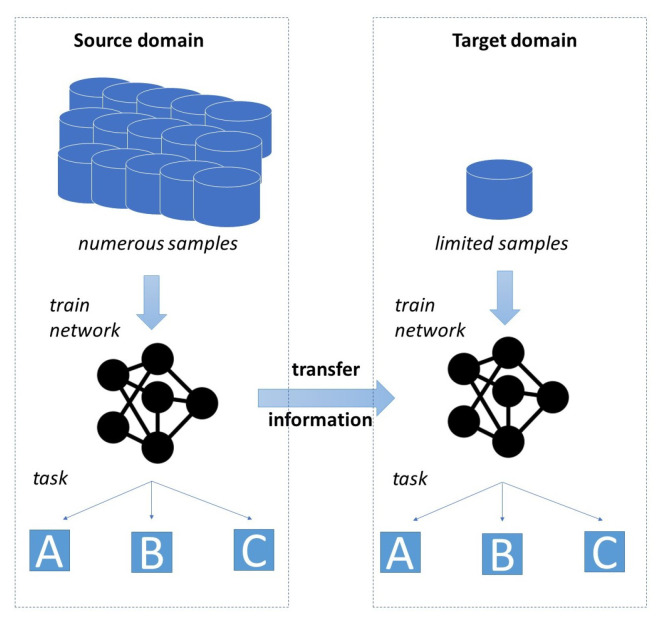
Illustration of the basic concept of transfer learning.

The biggest drawback of deep learning models is their heavy dependency on the availability of data. In particular, in many industrial environments, the collection and labeling of required data is expensive and time consuming. In turn, there are many settings where only a limited amount of training data are available. This widespread issue of data scarcity led to the emergence of research fields that aim to find approaches able to cope with a small amount of training data. Examples of corresponding approaches include few-shot learning, data augmentation, and transfer learning (TL). The concept of TL is illustrated by Figure 1. TL denotes the process of utilizing the information created when solving a task in order to solve a similar but distinct task.

In the context of deep learning, TL describes the pretraining of the layers of a network by a *source dataset* before transferring the learned parameters to a different model, the *target model*. Subsequently, the *target model* is retrained by the *target dataset*, which often comprises a small number of training samples (finetuning). As a basic idea, the information that the model learned by solving the *source problem* might be helpful to boost the training process of the *target problem*. Especially for production plants, TL constitutes a promising approach, as a high number of different product variants and models with widely varying quantities may be manufactured on the same shop floor. In particular, the data from products manufactured in high quantities might provide interesting information for similar goods with less turnover. This idea was recognized by many manufacturers, triggering a high number of publications in recent years about the successful use of TL in industrial settings (see, for example, [7,8]). Particularly in deep learning-based TSS, however, TL has received limited attention compared to its high prevalence in other areas like image recognition or condition monitoring. There is close to no research on applying TL approaches to TSS settings and on analyzing what factors increase or limit the benefits of TL. One of the very few contributions covering the topic showed promising results when applying cross-domain TL to boost medical TSS, even though the scope of the analysis concerning TL is very limited [9]. In general, no authors have ever performed a study focusing on how TL can be used to boost the performance of deep learning TSS models.

### 1.2. Our Contribution

With the presented problem setting and identified research gap in mind, this paper provides the following contributions:

- We analyze the benefits of using TL for TSS in an industrial setting. The paper provides one of the very first works to even tackle the problem of TL for TSS in general.
- We systematically analyze how pretraining with three different *source datasets* with varying degrees of similarity to the *target dataset* affects the performance of the *target model* after finetuning.
- We analyze to what degree the benefit of TL depends on the amount of available samples in the *target dataset*.
- The use case analyzed in the paper deals with the segmentation of operational states within the end-of-line testing cycle of hydraulic pumps. This is an innovative application of time series-based deep learning for a practical manufacturing problem.

The remainder of the paper is structured as follows: Section 2 provides an overview of related works in the area of industrial transfer learning for time series data. In Section 3, the concept of TL, the datasets used, and the experimental designs are presented. Section 4 describes the obtained experimental results and discusses them, followed by a conclusion and outlook in Section 5.

## 2. Literature Research

Facilitated by the general breakthrough of deep learning in many domains, the related research field of TL has recently received much attention and has advanced significantly. Different surveys were published in recent years, providing a detailed overview of the state of the art. The most extensive one focuses on the general concept of TL over all domains, including experimental results on text categorization and object detection [10]. More recently, a systematic study was published that had focused on TL in the context of time series data, discovering that the publication frequency has sharply increased since the year 2019 [11]. The study presented in [12] provides a detailed summary of TL concepts, use cases, and trends in the context of industrial automation. Another field that is highly related to TL and recently garnered significant attention is domain adaptation [13,14]. In this paper, however, we deal with a setting that exceeds the scope of domain adaptation (see Section 3.1) and, therefore, we do not cover the various frameworks from the field. Instead, we limit ourselves to reviewing relevant contributions in two areas, namely time series-based TL and deep industrial TL. We clarify that our literature review does not cover recent contributions in the field of domain adaptation.

### 2.1. Transfer Learning for Time Series

The concept of TL has its origin in the area of image recognition. After showing promising results there, it found its way into time series-based ML. The research field is still relatively young, with most contributions being published after 2018. However, despite the novelty of the research field, time series-based TL has already been applied in various fields like manufacturing [15], finance [16], geoscience [17], mobility [18], and medicine [19]. Successfully solved tasks include time series imaging [20], anomaly detection [21], classification [22], and forecasting [23]. A seminal work applying TL to time series classification analyzed how the pretraining of a model with different *source datasets* affects the classification accuracy of the *target task* [24]. In total, the authors tested more than 85 *source datasets* for pretraining and discovered that the benefit of pretraining significantly varies with the used *source dataset*. While a high similarity between *source dataset* and *target dataset* tended to lead to performance improvement, pretraining with dissimilar datasets did not improve or worsen the classification accuracy. Another interesting contribution demonstrates the successful application of TL to both convolutional neural networks (CNN) and long short-term memory (LSTM) architectures when performing time series classification and time series predictions [25].

Almost none of the previous works in the TL field focused on the task of TSS. While TSS problems might be less prevalent in the everyday life of companies and researchers, they still deserve attention due to their importance for fields like human action recognition [26], sleep staging [5], and operational state detection [6]. One contribution had a first look at the topic [9] and successfully showed that feature extraction process of a TSS–CNN model can be improved when pretraining the model with a large, domain-independent *source dataset*. However, a broad analysis focusing on TL and a detailed discussion of the implications of the results were not performed.

### 2.2. Deep Industrial Transfer Learning

Deep industrial transfer learning describes the concept of using formerly gained information and knowledge through deep learning to facilitate the solution of industrial problems [27]. In industrial settings, it is often not feasible (or even not possible) to accumulate a sufficiently large amount of data required for traditional deep learning approaches. The reasons for the data scarcity include the variability and high customization of processes and products [28], data protection regulations, and the high cost of data collection, storage, and labeling [29]. Industrial environments are highly volatile and dynamic. Wear, reconfigurations, and process adaptations [28] often diminish the value of historical data, leading to only a limited amount of short-term data accurately representing the correct relationship between input data and label [30]. Therefore, approaches like TL that attempt to address the issue of data scarcity have gained increasing attention in recent years.

Ref. [15] compared different TL approaches for anomaly detection based on pump vibration data. In turn, ref. [31] used already outdated data for pretraining their network to boost the prediction accuracy compared to a cold-start network. Results were verified on two industrial datasets (solar, furnace). Another approach combined both few-shot learning and TL techniques to overcome the issue of a small training set in rotatory machine fault detection; [7,8] used a huge general-domain dataset to train the feature extractor of a CNN, before finetuning the classificator with the small *target dataset* to diagnose gear faults. Ref. [32] analyzed whether TL can help to identify bearing defects by transferring learned features across data of different working conditions. The authors could show that their proposed TL approach increases the effectiveness of vibration-based fault detection models. Staying in the area of vibration-based bearing fault detection, another contribution provided an analysis of how and to what extent discriminative information found in varying settings (different sensor locations or working loads) can be transferred between networks [33]. In turn, ref. [34] investigated online fault detection of pumps and motor bearings and achieved high accuracy by creating a transfer convolutional neural network compared to classic models. Finally, ref. [35] was able to show that the deep TL network created with a domain adaptation module outperformed other approaches in a piston pump fault detection use case.

To the best of our knowledge, no other works have investigated the application of TL strategies to a TSS problem within an industrial setting. Almost all TL contributions from the time series domain focus on the tasks of forecasting, anomaly detection, and classification. There is a lack of research on TL strategies for TSS in general, and even more so for TSS use cases in industrial settings. The pump testing dataset we use in this paper was published in the context of one of our previous works [6]. In that work, the dataset was used for TSS experiments in which the availability of a sufficient amount of training data was ensured. In this paper, however, we deal with data scarcity and investigate whether TL can help to facilitate the learning of the model when few training samples are available. Therefore, we are the first to provide detailed results about the factors and conditions (sample size, *source dataset*, layer freezing) that determine the benefit of TL in the context of TSS. Potential explanations for why certain conditions favor TL are discussed as well. Our findings extend previous research about time series-based TL and provide insightful implications for practical industrial use cases.

## 3. Experimental Design and Data

### 3.1. Transfer Learning Formalization

**Figure 2:**
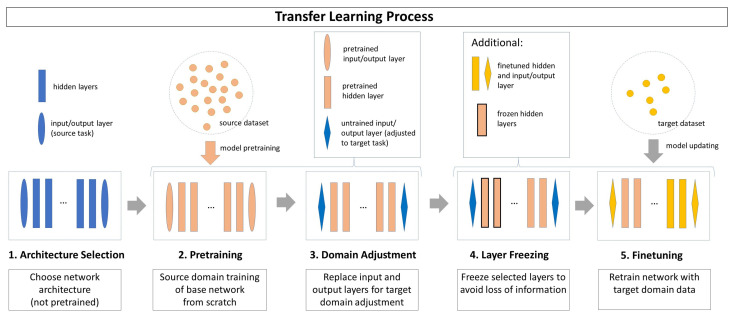
Visualization of 5-step transfer learning approach.

The idea of TL fundamentally differs from the traditional ML idea. Traditional ML approaches are isolation-based, meaning that they rely on an isolated dataset for the training of a distinct model in order to solve a single task. Usually, the models lack the ability to generalize well across different tasks or data. In turn, transfer learning allows for incorporating previously learned knowledge from other tasks in order to avoid some of the common ML drawbacks. Usually, the transferable knowledge is more valuable if the domains of the different models are related. While TL is a broad field with a number of different approaches and concepts, this paper focuses on a specific TL setting in which both the *source domain* and the *target domain* have labeled data and the transfer consists of pretraining a model with a *source dataset* (see Figure 2).

In our work, we reuse the formalization introduced by [36]. In general, an ML problem comprises two components, the domain and the task. A domain is formed by two elements, i.e., the feature space *X* and the probability distribution P(X). We denote a specific domain *D* as D=(X,P(X)). A task comprises two elements, namely the label space *Y* and the prediction function f(x) that is used to predict the label of a given instance *x*. The prediction function is learned by training the model via corresponding pairs of $x_{i}\in X$ and $y_{i}\in Y$. Moreover, function f(x) can be also interpreted as conditional probability distribution P(Y|X). Therefore, a specific task is denoted as T=(Y,P(Y|X)) or T=(Y,f(x)).

With these definitions, transfer learning can be formalized: Assume that we have a *source domain* $D_{s}$, a *source task*$T_{s}$, a *target domain*$D_{t}$, and a *target task* $T_{t}$. TL aims to enhance finding the most accurate *target model*f(x) in $T_{t}$ by incorporating the information in $D_{s}$ and $T_{s}$ with either $D_{s}\neq D_{t}$, or $T_{s}\neq T_{t}$, or both. One subcategory of TL is domain adaptation, which is applicable for scenarios in which only the probability distributions P(X) of $D_{s}$ and $D_{t}$ differ, while $T_{s}$ and $T_{t}$ are the same [37]. However, the experimental design of our paper does not include any setting in which *source task* $T_{s}$ and *target task* $T_{t}$ are equivalent. In turn, domain adaptation frameworks are not considered further.

The TL approach used in this paper is illustrated in Figure 2. It contains 5 steps:

- **Step 1 (Architecture Selection):** An untrained network architecture is selected, whose hidden layer structure is assumed to solve both the *source task* and the *target task*. The input and output layers are chosen to fit the *source task* and *source domain*.
- **Step 2 (Pretraining):** The network is trained with the *source domain dataset* on the *source task*. Usually, the *source dataset* is expected to contain a high number of samples to make the training effective.
- **Step 3 (Domain Adjustment):** The pretrained network is adjusted to the *target domain* and *target task*, whereas the knowledge found in its hidden layers is preserved. To achieve this, only the input and output layers are replaced by untrained layers adapting the network to the *target task* and *target domain*.
- **Step 4 (Layer Freezing):** Depending on the TL strategy, some or all hidden layers can be frozen before training for the *target task*. The parameters of a frozen layer are not updated in future training processes, which ensures that the knowledge learned during *source domain training* is preserved. As a drawback, the adaptation ability of the *target domain data* is limited.
- **Step 5 (Finetuning):** The network is retrained on the *target task* with the *target domain dataset*. Usually, the *target dataset* has only a limited number of instances. The resulting model includes information from both the *source domain* and the *target domain*.

**Figure 3:**
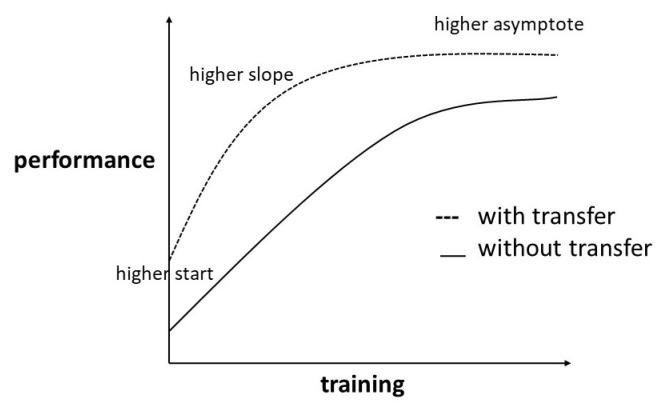
Three potential benefits of transfer learning.

The potential advantages of TL are illustrated by Figure 3. First, we obtain a better starting point for the model training compared to a cold start. This can be observed most often when *source domain* and *target domain* are heavily related and part of the learned knowledge is directly applicable to the *target task*. Second, a higher slope (faster learning process) can be achieved as the result of pretraining the hidden layers. By pretraining, parameters are often set in a generally functional way and just have to be finetuned for the new task. The most important advantage for industrial use cases is the higher asymptote, meaning a higher accuracy of the final model. This can often be observed in cases where the *target dataset* is very small and a part of the missing information in the *target dataset* can be filled by knowledge learned during the *source domain* training. We will observe which of the three benefits can be found in the different experimental settings we describe.

In this paper, we aim to analyze how pretraining with different *source datasets* affects the performance of the *target model*. To enable a high comparability, the selected *target dataset* and *target task* stay constant across all experiments. We define three different settings:

- **Setting 1:**  *Target domain* and *source domain* share the same feature space *X*, and *target task* and *source task* share the same label space *Y*. However, the domains differ in terms of probability distribution P(X) of the feature space, while the tasks differ in the feature–label relationship (conditional probability P(Y|X)).
- **Setting 2:** In addition to non-identical probability distributions P(X) and non-identical conditional probabilities P(Y|X), the label space *Y* of the *source task* and the *target task* differs as well. Only the feature space *X* of both domains is identical in this setting.
- **Setting 3:** All four elements (feature space *X*, label space *Y*, feature probability distribution P(X), and conditional probability P(Y|X)) differ between *source domain* and *target domain* as well as *source task* and *target task*.

### 3.2. Overview of Used Datasets

In the experiments, two datasets from two different domains were used, namely (1) the Hydraulic Pump End-of-Line dataset (HPEoL) and (2) the Opportunity dataset.

**(1)**  **Hydraulic Pump End-of-Line Dataset**

The Hydraulic Pump End-of-Line dataset collection was published recently. End-of-line testing is a type of manufacturing quality assurance process that is conducted at the end of the production line. It is used to ensure that the product meets the required specifications and that it is free from defects. In hydraulic pump testing, end-of-line testing usually consists of mounting the pump at a test bench and forcing it through a predefined testing cycle to check its function. The HPEoL dataset contains the end-of-line testing cycle data of around 198 hydraulic pumps measured by hydraulic and mechanical sensors on a hydraulic test bench. The dataset includes the testing cycle data of three different hydraulic pump types:

- Direct control pumps (DC): 120 instances distributed over three versions (V35, V36, V38) differing in size and technical specifications with 40 instances each.
- Speed-based (mechanical) control pumps (SC): 38 instances
- Proportional control pumps (PC): 40 instances

**Figure 4:**
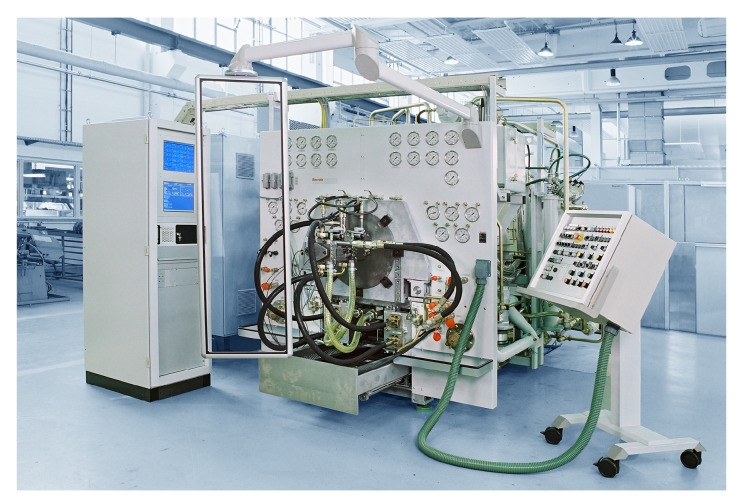
Hydraulic test bench with mounted pump and attached sensors (source: Bosch Rexroth AG).

For all control types, each sample corresponds to a multivariate time series with nine sensors representing the testing cycle of one specific pump. All 198 time series are multi-phased, meaning that each sample can be segmented into different segments representing the different testing phases of the testing cycle. While all phases of the testing cycle have to be completed for each pump, variations in the order of the phases and repetitions of phases are common. The data were collected with a frequency of 100 Hz. One testing cycle lasts between 3 and 7 min. Each time stamp has a integer-encoded state label representing the current testing phase of the pump at the time the sensor measures were taken. Supervised time series segmentation aims now to identify the different testing phases within the testing cycle by assigning state labels to all time stamps. Note that the testing cycles of the three different pump control types vary heavily (different label space *Y*), while the phases of different pump versions within a control type are very similar and only differ in the exact specification of a testing phase (identical label space *Y*, different conditional probabilities P(Y|X)). The number of different state labels for DC-types corresponds to 44, of PC-types to 43, and of SC-types to 44. Figure 4 depicts a hydraulic test bench.

**(2)**  **Opportunity Dataset**

**Figure 5:**
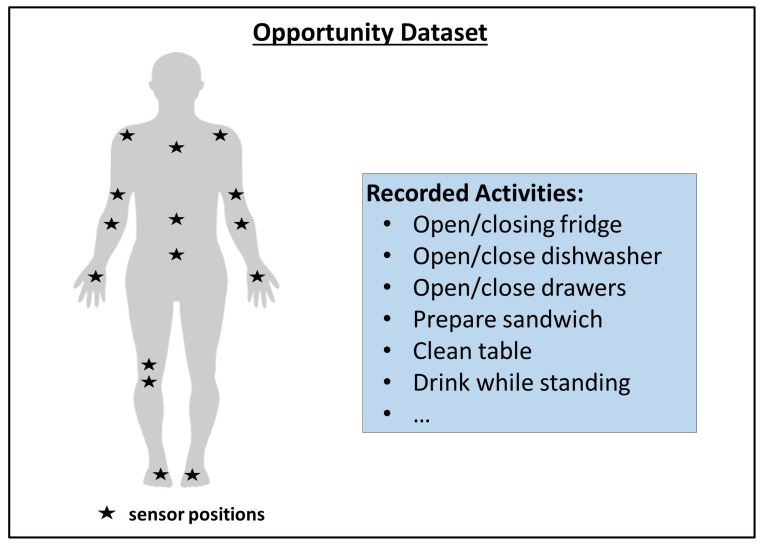
Opportunity dataset: Overview of sensor positions (adapted from [39]).

The Opportunity dataset was published to examine and compare human activity recognition algorithms, including TSS. It includes sequences created by tracking a number of human subjects via body-worn, object-attached, and ambient sensors (like accelerometers or location sensors). The sequences are annotated by activity labels. It is a popular and widely used dataset in the literature (e.g., [26,38]) that has often been used for benchmarking time series-based approaches. The dataset can be structured into two parts, i.e., the ADL (Activities of Daily Life) section and the Drill section. The ADL section just follows the subjects accomplishing daily life activities (including relaxing and eating) and, in turn, often has longer time periods without a change in the activity label. In the Drill section, however, the subjects were told to perform several short activities (e.g., open doors) without any breaks in between. The activity labels, therefore, are rapidly changing, resulting in a setting that is more similar to the HPEoL dataset, in which many label switches are present within a short period of time. Figure 5 shows the location of different sensors during the data collection process.

### 3.3. Model Architecture

**Figure 6:**
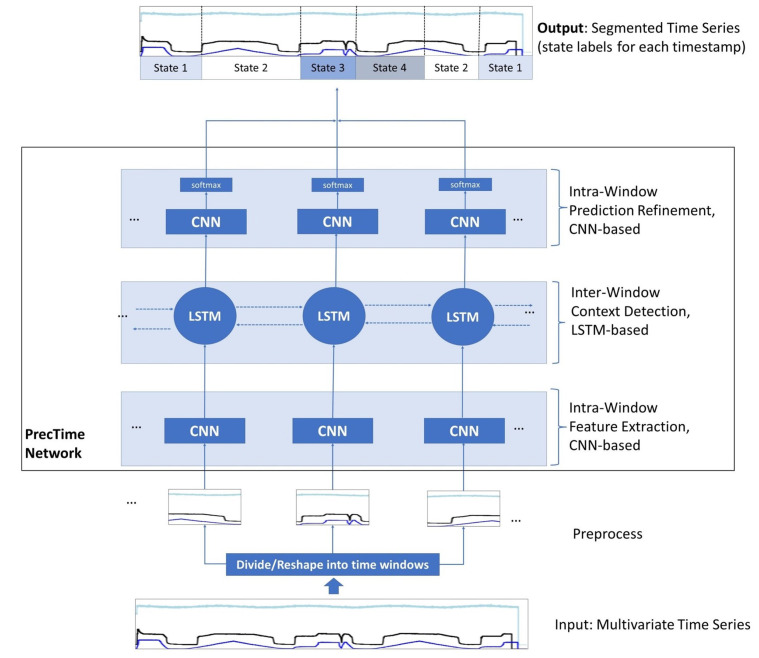
PrecTime architecture backbone with three modules (based on [6]).

All *source tasks* and *target tasks* investigated in this paper belong to the category of supervised TSS tasks. More precisely, we target to find and train a deep learning model to enable it to predict the state labels for all timestamps of a multivariate time series. This is called dense labeling. The network architecture we use to analyze the impact of TL in this work is called PrecTime. The PrecTime architecture was introduced in 2022 in one of our previous works [6]. It is designed for multivariate TSS and was already successfully applied to the HPEoL dataset and Opportunity dataset in isolation. As the output of the network is a sequence of state label predictions (one per time stamp), it is considered a sequence-to-sequence model. The conceptual backbone of the network is illustrated in Figure 6. PrecTime is a time window-based architecture consisting of three modules, i.e., a CNN-based intra-window feature extractor, an LSTM-based inter-window context detection, and a CNN-based intra-window prediction refinement model. The concrete details about the architecture, implementation, loss functions, and hyperparameters are described in [6]. They were reused in this paper.

### 3.4. Experimental Setup

**Figure 7:**
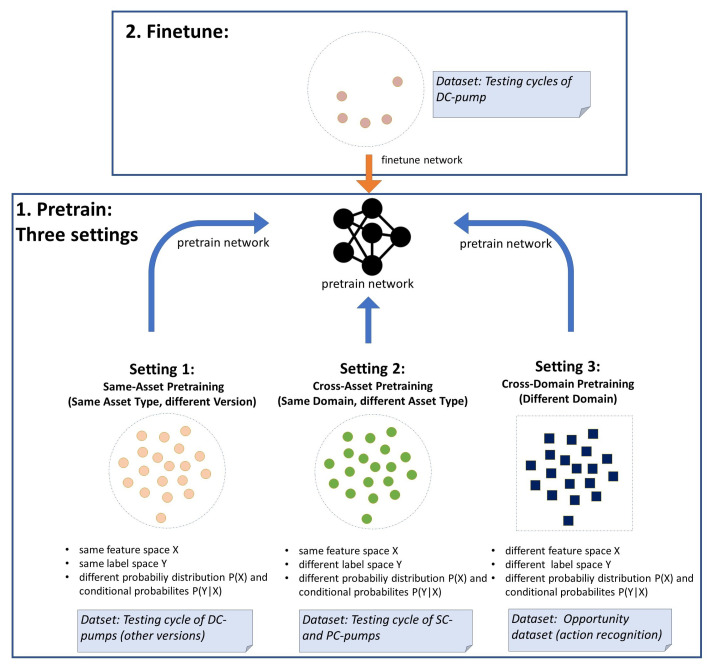
Overview of experimental setup with three settings.

The experimental setup consists of three settings with varying *source datasets* (see Figure 7). The *source datasets* were chosen in a way that three varying degrees of similarity between the *source dataset* and the *target dataset* are represented in the settings. The *target domains* and *target tasks* are kept constant over all experiments to enable direct comparability. In each setting, PrecTime is newly pretrained by the *source dataset* before being finetuned by the *target dataset*. As the amount of samples used for training might seem very low for deep learning, it has to be noted that in our TSS use case, each sample consists of several thousand labeled datapoints (one label per time stamp). Thus, a far lower number of samples is required to achieve good results compared to other deep learning tasks. The three settings are explained in detail in the following section.

**Setting 1 (Same-Asset Pretraining): Source data closely related to target data**

In the first setting, both *source datasets* and *target datasets* are taken from the HPEoL dataset. For the experiment, all three subsets belonging to the DC pump type are selected, with each of the subsets representing the 40 samples of a distinct DC-pump version (V35,V36,V38). The technical design of the three pump versions differs only slightly. In turn, the testing cycles of the three pumps are very similar as well. Only detailed specifications of certain testing phases may vary. Based on the high similarity, we call this setting same-asset pretraining. Using the formalization introduced in Section 3.1, we have a setting where the feature space *X* and label space *Y* of *source data* and *target data* are identical, while the probability distribution P(X) and conditional probabilities P(Y|X) slightly differ. In the experiments, each pump version (V35,V36,V38) is used once as the *target dataset*, while the data of the remaining two versions are merged to become the combined *source dataset*. For each of these three scenarios, we perform various experimental runs, in which we limit the sample number in the *target training set* to 1, 3, 5, and 10. This allows us to investigate how the number of samples in the *target training dataset* affects the benefit of pretraining considering training speed and accuracy. Furthermore, two layer freezing approaches are compared. In the first approach, all layers except the output layer were frozen (TL-fr). In the second approach, all layers were set as trainable (TL-tr). A non-pretrained model that was trained only by the *target dataset* is defined as the baseline (BL1). Additionally, as the feature space *X* and label space *Y* are identical, an additional baseline can be created by evaluating the model performance when merging the data of all three pump versions into one big training set (BL2). Note that this additional baseline is only available in Setting 1, as it requires equivalence of feature space and label space.

**Setting 2 (Cross-Asset Pretraining): Source data distantly related to target data**

As in Setting 1, all *source datasets* and *target datasets* are subsets of the HPEoL dataset. However, in this case, all three pump types (DC, SC, and PC) are considered. Each of the three DC pump versions (V35,V36,V38) is used as the *target data*, while both the SC pump subset and the PC pump subset are distinctly used as *source data*. The testing cycles of the different control types (DC, SC, and PC) differ heavily. However, the used test benches and sensors are identical across all types. Therefore, we have still have an identical feature space *X* across *source data* and *target data* in this setting. We limit the *target domain* training data to three instances to make the impact of TL strongly visible. The TL is performed with all layers unfrozen. A non-pretrained model is set as the baseline (BL1).

**Setting 3 (Cross-Domain Pretraining): Source data non-related to target data**

In Setting 3, it is explored whether pretraining with a non-related *source dataset* can boost the accuracy of the *target task*. As in Settings 1 and 2, the three DC pump versions are distinctly chosen as *target sets*. In this case, however, the pretraining is not done by HPEoL subsets, but by the Opportunity dataset (Section 3.1). The Opportunity dataset contains sensor location and movement data of human subjects and therefore covers a completely different domain compared to pump testing. As in setting two, the *target domain* training data are limited to three instances, and the TL is performed with all layers unfrozen. Again, the non-pretrained model BL1 is set as the baseline.

### 3.5. Implementation Details

The networks were trained on an *NVIDIA Tesla K80* with 6 cores, 56 GB RAM, and 380 GB disk storage in the *Microsoft Azure* cloud. The implementation was done with *Python*, mainly utilizing the *Tensorflow* and *Keras* packages. For the pretraining, all available samples found in the selected *source dataset* were used. The *target dataset* was split into a training set and a validation set. The size of the *target training dataset* was set to a predefined limited size in each Setting (see Section 4.3). The training sample size in Setting 1 was varied in different experimental runs (1, 3, 5, 10), while the training sample size in Settings 2 and 3 was kept constant as 3. To achieve comparability, the *target validation set* was kept constant across all settings. It was defined to include 20 samples from the *target dataset*. The evaluation of the model performance was done by tracking the progress of the validation accuracy for 100 epochs. The accuracy was calculated by dividing the number of time stamps that were correctly labeled by the mode by the total number of time stamps. In pretraining, the batch size was defined as 20, while during finetuning, it was set equal to the number of training samples. Cross-entropy was chosen as the loss function to be optimized during the training process. The window size was defined to be 100 in all experiments. The preprocessing included using zero-padding to bring all instances to a fixed length (45,000 for HPEoL, 60,000 for opportunity). For the Opportunity dataset only the drill-section data were used as samples, and 113 sensors were selected as is the common practice in the literature [26]. Missing sensor values were interpolated.

## 4. Results and Discussion

The following section gives detailed insight into the experimental results and also discusses them.

### 4.1. Results for Setting 1: Same-Asset Pretraining

Table 1 provides an overview of the achieved validation accuracies in Setting 1. It can be clearly seen that the TL-fr approach (hidden layers set frozen) showed a very weak performance. Thus, the approach seems to be the wrong one for the use case of supervised TSS. In turn, we focus our analysis on TL-tr (all layers unfrozen), which showed a very strong performance in comparison. Pretraining with the *source dataset* from the same asset type increased the final accuracy of the network in all experiments. A clear negative correlation between the amount of available *target data* and the benefit of TL can be observed, meaning TL shows its greatest potential when facing *target data* scarcity. While the exact numbers differ in detail, the observations are consistent across all three DC pump versions. When having a *target training set* of size one, the accuracy difference between the unfrozen TL-tr approach and the isolated training baseline (BL1) ranged from 10% for the V38 pump to 34% for the V35 pump. On the contrary, in the experimental runs using the highest number of *target training samples* (10), the performance boost decreased to around 2–3%, which is helpful but far less significant. Interestingly, no clear conclusion could be drawn about the benefit of combining *target data* and *source data* into one large training set and training the network with it (BL2). While in some experimental runs, the combined training led to accuracy improvement compared to isolated *target data* training (BL1), in other cases it seemed to impede model performance. However, note that combining *source data* and *training data* into one big dataset (BL2) in no case showed a better performance than the TL-tr approach, emphasizing the superiority of using TL in this setting.

**Figure 8:**
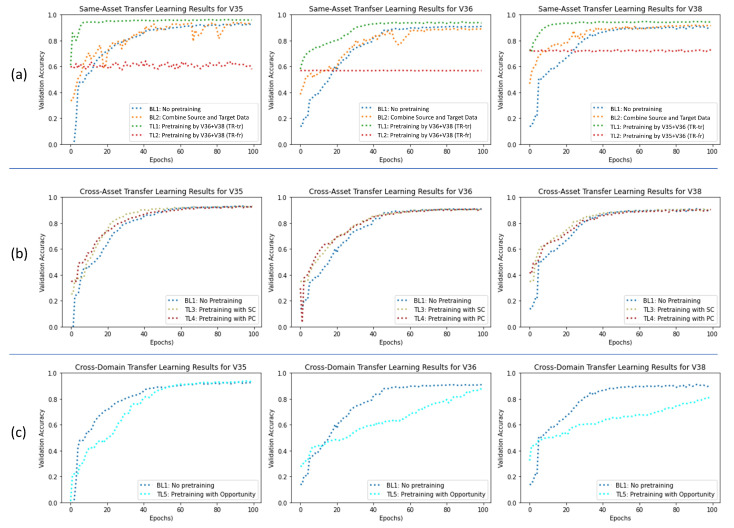
Training process comparison for different settings, all with a target training set of size 3: (**a**) Setting 1: Same-asset transfer learning T1 (unfrozen) and T2 (frozen) compared to two baselines BL1 and BL2, (**b**) Setting 2: Cross-asset transfer learning T3 (SC pretraining) and T4 (PC pretraining) within same domain compared to baseline BL1, (**c**) Setting 3: Cross-domain transfer learning T5 (Opportunity pretraining) compared to baseline BL1.

Figure 8a shows the training process of all three pump versions for a *target training set* of size 3. Clearly, we can observe that for unfrozen TL (TL-tr), not only is the final accuracy improved (higher asymptote), but also the training speed is significantly increased. Very high accuracies (>90%) are reached far earlier compared to the two baselines (BL1, BL2). The main reason for the increased training speed seems to be not a steeper slope, but a higher starting point achieved through pretraining. The extent of this observation varies across the three pumps; it is most visible in the V35 training process. The high starting points after pretraining seem to be the result of using *source data* and *training data* of high similarity. For frozen TL, it becomes obvious that freezing all hidden layers almost completely eliminates any training progress in this setting. The models end up with almost the same performance before and after finetuning (TL-fr).

### 4.2. Results for Setting 2: Cross-Asset Pretraining

In Setting 2 (pretraining by *source data* from a different asset type), the benefits of TL are diminished clearly compared to Setting 1 (see Table 2). When examining the final model performance, no clear differences (>1%) across the three pretraining scenarios can be observed for any of the three DC pump versions. Therefore, the potential TL benefit of a higher asymptote is not observed in this setting.

However, when taking a closer look at the training process in Figure 8b, we can make an insightful observation. Despite all model training processes ending on a similar performance level after 100 epochs, in the first half of the training process, the models with applied TL show a better performance compared to the models without pretraining. This difference disappears more and more in later training stages. This can be explained by the fact that the pretrained models avoid a complete cold start and the pretraining has already set parameters in a certain way that helps the *target data* training process to progress quicker. Despite the non-identical label space of the *source data* and *target data*, the pretrained models seem to benefit from a certain relatedness in the nature of the data (shared feature space) at the start of the training.

### 4.3. Results for Setting 3: Cross-Domain Pretraining

As opposed to the observed benefits of TL in Settings 1 and 2, we can observe a form of negative TL in Setting 3. As the *source dataset* (Opportunity) in this setting belongs to a completely different domain than the *target dataset* (HPEoL), pretraining does not lead to any benefit, but instead impairs the training speed of the *target data* (see Figure 8c). The different nature of the data prevents a meaningful pretraining that can be exploited later by the *target task*. However, despite the slowed training process, the final asymptote level of the pretrained network (TL5) is similar to or only slightly below that of the non-pretraining scenario (BL1) for all three pump variants. This can be explained by the fact that all layers were set to trainable. Thus, the pretrained models are able to overcome their impaired starting point caused by cross-domain pretraining over the course of the training process and fully adapt to the new domain.

Altogether, the findings provide systematic evidence for the hypothesis that the benefit of TL in TSS is (1) increasing with lack of *target training data* and (2) increasing the similarity between *source data* and *target data*. Table 3 summarizes the observed results. While we assume these results are to some extent transferable to other domains, further research has to be done to confirm the general validity of our finding.

While freezing layers is often recommended in the literature, in this work, it heavily aggravated the model performance in initial experiments (see TL-fr results in Setting 1) and was therefore not investigated deeper. However, checking different layer freezing strategies in a more nuanced way might be an interesting direction to go for future research.

## 5. Conclusions

We performed a systematic investigation of different TL scenarios and strategies for an industrial TSS problem. It was examined whether and how pretraining with close- to non-related *source datasets* improves the network learning performance of PrecTime, a sequence-to-sequence deep learning architecture for TSS. Focus points were the speed of the training process and the achieved validation accuracy after completing the training. The *target task* was specified to be the supervised segmentation of hydraulic pump testing cycles into different testing phases via the prediction of state labels. Three different *source datasets* were selected for pretraining (1) different versions of same pump type, (2) different pump types, and (3) different domains. It was observed that pretraining with a closely-related *source dataset* led to the biggest benefit (Setting 1), while pretraining with a *source dataset* from a different domain even aggravated the training process (Setting 3). Additionally, we could show that TL performed best in comparison to baselines in scenarios in which the *target training dataset* had the smallest sample size.

The main limitation of our study is the focus on a specific use case. While our results provide interesting findings, the domain-independence and general validity of the results still have to be established. Furthermore, the important topic of layer freezing was not investigated in depth. Therefore, future work could look deeper into different layer freezing strategies and aim to provide recommendations as to which layers should be frozen and which layers set to trainable. Additionally, one could check if incrementally unfreezing layers over the course of the training process leads to promising results. Possible research directions could also include performing experiments with different TSS network architectures and datasets to validate and build on the findings of this paper. It could also be examined how pretraining with multiple datasets or a general-domain dataset affects the model performance. Finally, the transfer of knowledge between labeled data and unlabeled data could be investigated in the context of TSS.

## Figures and Tables

**Table 1:** Validation accuracies (%) after 100 epochs in Setting 1 for BL1 (no pretraining), BL2 (combine source and target dataset), TL-fr (layers frozen), and TL-tr (layers unfrozen).

Number of Training Samples in Target Data	V35 as Target Data V36 + V38 as Source				V36 as Target Data V35 + V38 as Source				V38 as Target Data V35 + V36 as Source			
	**BL1**	**BL2**	**TL-fr**	**TL-tr**	**BL1**	**BL2**	**TL-fr**	**TL-tr**	**BL1**	**BL2**	**TL-fr**	**TL-tr**
1	59.0	45.2	53.0	93.8	66.9	45.0	50.3	86.0	77.1	83.9	69.2	88.7
3	92.3	92.5	57.8	95.9	90.9	88.9	56.8	93.5	90.2	91.3	73.0	94.4
5	94.3	92.5	53.5	96.9	92.5	92.5	60.2	95.5	90.4	93.1	70.7	95.8
10	95.7	97.0	58.4	97.4	94.1	95.2	59.3	96.7	93.1	96.3	69.0	96.5

**Table 2:** Validation accuracies (%) after 100 epochs in Setting 2 for BL1, SC-, and PC-pretraining.

Setting	V38 as Target Dataset	V36 as Target Dataset	V35 as Target Dataset
BL1 (no pretraining)	90.2	91.0	92.4
Pretraining by SC pump dataset	90.8	90.5	92.9
Pretraining by PC pump dataset	90.4	90.8	92.4

**Table 3:** Summary table of observed TL effects (target training set of size 3, all layers unfrozen) in the three experimental settings (++: very positive effect, +: positive effect, 0: negligible effect, -: negative effect).

Setting No.	Setting Description	Effect on Asymptote	Effect on Training Start	Effect on Training Slope
1	Same-asset pretraining (closely related source and target data)	+	++	+
2	Cross-asset pretraining (distantly related source and target data)	0	+	+
3	Cross-domain pretraining (non-related source and target data)	0	0	-

## Data Availability

The Hydraulic Pump End-of-Line dataset is accessible via https://github.com/boschresearch/Hydraulic-EoL-Testing/ (accessed on 22 February 2023). The Opportunity dataset is accessible via https://archive.ics.uci.edu/ml/datasets/opportunity+activity+recognition (accessed on 22 February 2023).

## Abbreviations

The following abbreviations are used in this manuscript:

ADL	activities of daily life
BL1	baseline 1
BL2	baseline 2
CNN	convolutional neural network
DC	direct control
HPEoL	hydraulic pump end-of-line
LSTM	long short-term memory
ML	machine learning
PC	proportional control
SC	speed-based control
TL	transfer learning
TSS	time series segmentation

## References

[1] Kaveh A., Talatahari S., Khodadadi N. (2022). Stochastic Paint Optimizer: Theory and application in civil engineering. Eng. Comput..

[2] Hoppenstedt B., Pryss R., Stelzer B., Meyer-Brötz F., Kammerer K., Treß A., Reichert M. (2018). Techniques and Emerging Trends for State of the Art Equipment Maintenance Systems—A Bibliometric Analysis. Appl. Sci..

[3] Hoppenstedt B., Reichert M., Kammerer K., Probst T., Schlee W., Spiliopoulou M., Pryss R. (2019). Dimensionality Reduction and Subspace Clustering in Mixed Reality for Condition Monitoring of High-Dimensional Production Data. Sensors.

[4] Kammerer K., Hoppenstedt B., Pryss R., Stökler S., Allgaier J., Reichert M. (2019). Anomaly Detections for Manufacturing Systems Based on Sensor Data–Insights into Two Challenging Real-World Production Settings. Sensors.

[5] Phan H., Andreotti F., Cooray N., Chén O., de Vos M. (2018). SeqSleepNet: End-to-End Hierarchical Recurrent Neural Network for Sequence-to-Sequence Automatic Sleep Staging. IEEE Trans. Neural Syst. Rehabil. Eng..

[6] Gaugel S., Reichert M. PrecTime: A Deep Learning Architecture for Precise Time Series Segmentation in Industrial Manufacturing Operations, PrePrint. www.academia.edu.

[7] Lu N., Hu H., Yin T., Lei Y., Wang S. (2021). Transfer Relation Network for Fault Diagnosis of Rotating Machinery with Small Data. IEEE Trans. Cybern..

[8] Cao P., Zhang S., Tang J. (2018). Preprocessing-Free Gear Fault Diagnosis Using Small Datasets With Deep Convolutional Neural Network-Based Transfer Learning. IEEE Access.

[9] Matias P., Folgado D., Gamboa H., Carreiro A. (2021). Time Series Segmentation Using Neural Networks with Cross-Domain Transfer Learning. Electronics.

[10] Zhuang F., Qi Z., Duan K., Xi D., Zhu Y., Zhu H., Xiong H., He Q. (2021). A Comprehensive Survey on Transfer Learning. Proc. IEEE.

[11] Weber M., Auch M., Doblander C., Mandl P., Jacobsen H.A. (2021). Transfer Learning With Time Series Data: A Systematic Mapping Study. IEEE Access.

[12] Maschler B., Weyrich M. (2021). Deep Transfer Learning for Industrial Automation: A Review and Discussion of New Techniques for Data-Driven Machine Learning. IEEE Ind. Electron. Mag..

[13] Li W., Gao H., Su Y., Momanyi B.M. (2022). Unsupervised Domain Adaptation for Remote Sensing Semantic Segmentation with Transformer. Remote Sens..

[14] Liu X., Yoo C., Xing F., Oh H., Fakhri G., Kang J.W., Woo J. (2022). Deep Unsupervised Domain Adaptation: A Review of Recent Advances and Perspectives. APSIPA Trans. Signal Inf. Process..

[15] Heistracher C., Jalali A., Strobl I., Suendermann A., Meixner S., Holly S., Schall D., Haslhofer B., Kemnitz J. Transfer Learning Strategies for Anomaly Detection in IoT Vibration Data. Proceedings of the IECON 2021—47th Annual Conference of the IEEE Industrial Electronics Society, IEEE.

[16] He Q.Q., Pang P.C.I., Si Y.W. Multi-source Transfer Learning with Ensemble for Financial Time Series Forecasting. Proceedings of the 2020 IEEE/WIC/ACM International Joint Conference on Web Intelligence and Intelligent Agent Technology (WI-IAT).

[17] Yan J., Wang L., He H., Liang D., Song W., Han W. (2022). Large-Area Land-Cover Changes Monitoring With Time-Series Remote Sensing Images Using Transferable Deep Models. IEEE Trans. Geosci. Remote Sens..

[18] Lian R., Tan H., Peng J., Li Q., Wu Y. (2020). Cross-Type Transfer for Deep Reinforcement Learning Based Hybrid Electric Vehicle Energy Management. IEEE Trans. Veh. Technol..

[19] Aldayel M.S., Ykhlef M., Al-Nafjan A.N. (2020). Electroencephalogram-Based Preference Prediction Using Deep Transfer Learning. IEEE Access.

[20] Gross J., Buettner R., Baumgartl H. (2022). Benchmarking Transfer Learning Strategies in Time-Series Imaging: Recommendations for Analyzing Raw Sensor Data. IEEE Access.

[21] Wen T., Keyes R. (2019). Time Series Anomaly Detection Using Convolutional Neural Networks and Transfer Learning. arXiv.

[22] Gikunda P., Jouandeau N. Homogeneous Transfer Active Learning for Time Series Classification. Proceedings of the 2021 20th IEEE International Conference on Machine Learning and Applications (ICMLA).

[23] Warushavithana M., Mitra S., Arabi M., Breidt J., Pallickara S.L., Pallickara S. A Transfer Learning Scheme for Time Series Forecasting Using Facebook Prophet. Proceedings of the 2021 IEEE International Conference on Cluster Computing (CLUSTER).

[24] Fawaz H.I., Forestier G., Weber J., Idoumghar L., Muller P.A. Transfer learning for time series classification. Proceedings of the 2018 IEEE International Conference on Big Data (Big Data).

[25] Dridi A., Afifi H., Moungla H., Boucetta C. Transfer Learning for Classification and Prediction of Time Series for Next Generation Networks. Proceedings of the ICC 2021—IEEE International Conference on Communications.

[26] Yao R., Lin G., Shi Q., Ranasinghe D. (2018). Efficient Dense Labeling of Human Activity Sequences from Wearables using Fully Convolutional Networks. Pattern Recognit..

[27] Maschler B., Vietz H., Jazdi N., Weyrich M. Continual Learning of Fault Prediction for Turbofan Engines using Deep Learning with Elastic Weight Consolidation. Proceedings of the 2020 25th IEEE International Conference on Emerging Technologies and Factory Automation (ETFA).

[28] Kammerer K., Pryss R., Reichert M. Context-Aware Querying and Injection of Process Fragments in Process-Aware Information Systems. Proceedings of the 2020 IEEE 24th International Enterprise Distributed Object Computing Conference (EDOC).

[29] Maschler B., Knodel T., Weyrich M. Towards Deep Industrial Transfer Learning for Anomaly Detection on Time Series Data. Proceedings of the 2021 26th IEEE International Conference on Emerging Technologies and Factory Automation (ETFA).

[30] Tercan H., Guajardo A., Meisen T. Industrial Transfer Learning: Boosting Machine Learning in Production. Proceedings of the 2019 IEEE 17th International Conference on Industrial Informatics (INDIN), IEEE.

[31] Zhou X., Zhai N., Li S., Shi H. (2022). Time Series Prediction Method of Industrial Process with Limited Data Based on Transfer Learning. IEEE Trans. Ind. Inform..

[32] Xu W., Wan Y., Zuo T.Y., Sha X.M. (2020). Transfer Learning Based Data Feature Transfer for Fault Diagnosis. IEEE Access.

[33] Wu S., Jing X.Y., Zhang Q., Wu F., Zhao H., Dong Y. (2020). Prediction Consistency Guided Convolutional Neural Networks for Cross-Domain Bearing Fault Diagnosis. IEEE Access.

[34] Xu G., Liu M., Jiang Z., Shen W., Huang C. (2020). Online Fault Diagnosis Method Based on Transfer Convolutional Neural Networks. IEEE Trans. Instrum. Meas..

[35] He Y., Tang H., Ren Y. A Multi-channel Transfer Learning Framework for Fault Diagnosis of Axial Piston Pump. Proceedings of the 2021 Global Reliability and Prognostics and Health Management (PHM-Nanjing).

[36] Lin Y.P., Jung T.P. (2017). Improving EEG-Based Emotion Classification Using Conditional Transfer Learning. Front. Hum. Neurosci..

[37] Sun S., Shi H., Wu Y. (2015). A survey of multi-source domain adaptation. Inf. Fusion.

[38] Chambers R.D., Yoder N.C. (2020). FilterNet: A Many-to-Many Deep Learning Architecture for Time Series Classification. Sensors.

[39] Chavarriaga R., Sagha H., Bayati H., Millan J.d.R., Roggen D., Förster K., Calatroni A., Tröster G., Lukowicz P., Bannach D. Robust activity recognition for assistive technologies: Benchmarking machine learning techniques. Proceedings of the Workshop on Machine Learning for Assistive Technologies at the Twenty-Fourth Annual Conference on Neural Information Processing Systems (NIPS-2010).

